# An Analysis of the Influence of Selected Genetic and Hormonal Factors on the Occurrence of Depressive Symptoms in Late-Reproductive-Age Women

**DOI:** 10.3390/ijerph120403547

**Published:** 2015-03-27

**Authors:** Anna Jurczak, Małgorzata Szkup, Agnieszka Samochowiec, Anna Grzywacz, Jerzy Samochowiec, Beata Karakiewicz, Barbara Dołęgowska, Elżbieta Grochans

**Affiliations:** 1 Department of Nursing, Pomeranian Medical University in Szczecin, Żołnierska Str. 48, 71-210 Szczecin, Poland; E-Mails: jurczaka@pum.edu.pl (A.J.); m.szkup@onet.eu (M.S.); 2 Department of Clinical Psychology, Institute of Psychology, University of Szczecin, ul. Krakowska 71–79, 71-017 Szczecin, Poland; E-Mail: samoaga@tlen.pl; 3 Department of Psychiatry, Pomeranian Medical University in Szczecin, Broniewskiego Str. 26, 71-460 Szczecin, Poland; E-Mails: annagrzywacz@gazeta.pl (A.G.); samoj@pum.edu.pl (J.S.); 4 Public Health Department, Pomeranian Medical University in Szczecin, Żołnierska Str. 48, 71-210 Szczecin, Poland; E-Mail: karabea@sci.pum.edu.pl; 5 Laboratory of Stem Cell Physiology and Biochemistry, Division and Department of Physiology, Pomeranian Medical University in Szczecin, Żołnierska Str. 48, 71-210 Szczecin, Poland; E-Mail: barbara.dolegowska@pum.edu.pl; 6 Department of Nursing, Pomeranian Medical University in Szczecin, Żołnierska Str. 48, 71-210 Szczecin, Poland

**Keywords:** polymorphism, AMH, FSH, late reproductive period, depressive symptoms, 5-HTTLPR, *MAO-A*

## Abstract

*Background*: The aim of this study was to analyze the influence of genetic and hormonal factors on incidences of depressive symptoms in late-reproductive-age women. *Methods*: The study was performed using the Beck Depression Inventory, the PCR, and genetic tests of 347 healthy late-reproductive-age Polish women. *Results*: The relationship between the level of anti-Müllerian hormone (AMH) and depressive symptoms was not statistically significant (*p* > 0.05). Increases in age and FSH levels were accompanied by a decrease in AMH level in a significant way (*p* < 0.05). There were no statistically significant relationships between the distribution of genotypes and the frequency of alleles of the investigated polymorphisms and depressive symptoms according to the Beck Depression Inventory. *Conclusions*: (1) The presence of the *s/s genotype* of the *5-HTTLPR* polymorphism in the serotonin transporter promoter region and the 3/3 genotype of the *30-bp VNTR* polymorphism in the monoamine oxidase A promoter region does not contribute to the development of depressive symptoms in late-reproductive-age women. (2) A relationship between the level of anti-Müllerian hormone and depressive symptoms was not confirmed in the group of healthy late-reproductive-age women. (3) AMH level correlates negatively with FSH level and age, which confirms that AMH can be regarded as a factor reflecting the ovarian reserve.

## 1. Introduction

In accordance with the definition proposed by the Stages of Reproductive Aging Workshop (STRAW) in 2001, the life of an adult woman can be divided into reproductive, menopausal transition and postmenopausal periods. The reproductive period is characterized by the occurrence of three stages: the early (‒5), peak (‒4), and late (‒3) stages. The latter, which is analyzed in this study, marks the time when fecundity begins to decline. At this stage, the first subtle changes in menstrual cycles can be observed, among them endocrine changes that have effects on the woman’s fertility. According to the STRAW + 10 recommendations, this period should be divided into ‒3b and ‒3a subphases. In the ‒3b phase, the length of the menstrual cycle and FSH level remain unchanged, but the level of AMH and antral follicle count decrease. The ‒3a phase is characterized by a shortening of the duration of the menstrual cycle. The FSH level in the early follicular phase considerably increases, whereas the levels of other ovarian markers decrease [1]. 

The last two decades of the twentieth century produced many studies, mostly cross-sectional, which served as a basis for drawing often contradictory conclusions, either confirming or denying the relationship between perimenopause and an increase in depressive symptoms in women [2,3]. Most reports support the hypothesis that sex hormones contribute to mood and anxiety disorders. Nevertheless, the psychopathological mechanism underlying this relationship has not yet been fully explained [4]. The levels of sex hormones, which have an impact on women’s brain function, change after menopause, possibly contributing to the development of neurodegenerative diseases and mental disorders, as has been confirmed by studies of the brains of perimenopausal and postmenopausal women conducted using magnetic resonance, positron emission computed tomography, and single photon emission tomography [5]. A review of functional magnetic resonance imaging (fMRI) studies of the influence of sex hormones on emotional and cognitive functioning demonstrated that steroid sex hormones play a role in cerebral cortical and subcortical regions [6]. The fMRI study carried out by Frey *et al.* among 11 women aged 40–60 years revealed a more significant engagement of the dorsolateral prefrontal cortex and less activation of the amygdala in emotional regulation in midlife women than in younger ones [7]. A study conducted on the macaque model showed that ovarian steroids increase the cellular resilience of serotonin neurons and may prevent serotonin neuron death after menopause. The survival of serotonin neurons is essential for mental health and cognitive processes [8]. It remains unclear how decreasing levels of sex hormones during perimenopause influence the development of depressive symptoms. Estrogen plays an important role in the system of neurotransmitters, and so a decline in its level during perimenopause and menopause not only entails a worsening of the cognitive function, but also contributes to a higher risk of depressive disorders. Many longitudinal studies indicate that the risk of depressive symptoms is significantly higher in the perimenopausal period than in the late reproductive period [9]; this may be associated with changes in hormone levels. In Freeman’s study [10], increased odds of severe depressive symptoms were related to the levels of follicle-stimulating hormone (FSH). FSH is a glycoprotein hormone that plays an essential role in the development and maturation of ovarian follicles and the secretion of gonadal hormones [11]. The increase in follicular phase FSH prior to menopause is attributed to an early decline in the ovarian hormone inhibin B, which negatively regulates its secretion [12]. Prior *et al.* [13] question the predictive value of FSH and inhibin B levels for the onset of menopause. At the same time, they mention another marker that can be used to assess the age of ovaries, anti-Müllerian hormone (AMH), speculating that it may be a more effective predictor. AMH, which has only been investigated for a relatively short time, is a dimeric glycoprotein, a member of the transforming growth factor (TGF)-β superfamily [14]. 

The development of depression in the perimenopausal period may be determined by psychological, biological, and social factors, but its mechanism has not yet been clearly defined. At this stage of life, many women experience problems caused by fluctuating levels of estrogen, including vasomotor symptoms, vaginal dryness, poor sleep, and depressed mood [15]. Numerous studies point to the influence of abnormalities in the functioning of the monoaminergic system on the pathogenesis of depressive disorders [16]. According to Newport, a monoamine imbalance may contribute to the development of depressive symptoms in women [17]. Monoamine oxidase *A (MAO-A)* inhibitors and the serotonin transporter gene (*SLC 6A4*) are recognized as having the most profound effects on the functioning of the monoamine neurotransmitter system. The expression of these genes depends on the type of the polymorphism, which may determine the occurrence of specific mood disorders [16]. *MAO-A* causes oxidative stress, contributes to apoptosis, and metabolizes monoamines, which may lead to mood disorders and dementia. Studies show that the total *MAO-A* total distribution volume in perimenopausal women is 34% higher than in reproductive age women, especially in the prefrontal cortex, which is positively correlated with tendency to cry [18].

*5HTT* is an integral membrane protein that moves the serotonin neurotransmitter from the synaptic cleft to the presynaptic neurons. *5HTT* is encoded by a single gene on the 17q12 chromosome. A functional serotonin-transporter-linked polymorphic region (5-HTTLPR) polymorphism is characterized by the insertion or deletion of a 44-bp fragment and, consequently, the creation of a short or a long allele, associated with different gene transcriptional activity. A short allele has a lower ability to uptake serotonin than a long allele [19]. *MAO-A* contributes to the development of depression, because it is involved in the degradation of monoamines, such as dopamine, serotonin, and noradrenaline [20]. The *MAO-A* gene may be also responsible for an inclination to depression. Sabol *et al.* were the first to describe the *MAO-A* polymorphism, which is a variable-number tandem repeat (*VNTR*) polymorphism in the *MAO-A* promoter region [21]. It consists of a 30-bp repeated sequence that may be present in 3, 3.5, 4, or 5 copies [22]. The 3R allele is associated with a lower gene transcriptional activity, while the 3.5R, 4R, and 5R alleles are related to a higher *MAO-A* activity [23]. The research carried out by Sabol and Deckert revealed significant differences in enzyme activity. It was observed that alleles with 3.5 or 4 copies of the repeated sequence are transcribed more efficiently than the ‘3’ allele in the *in vitro* examination. For 5R alleles, the research results were ambiguous [21,24]. 

Bearing in mind that AMH and FSH have been identified as predictors of ovarian aging [1], we attempted in the present study to assess the influence of changes in the levels of these hormones on the severity of depressive symptoms among late-reproductive-age women. Additionally, based on genetic testing, we analyzed the women’s predisposition to depressive symptoms in relation to the presence of *5HTT* and *MAO-A* gene polymorphisms.

The aim of this study was to analyze:

1. The influence of genetic factors such as the presence of the *44-bp VNTR* polymorphism in the *5HTT (SLC 6A4)* promoter region, and the *30-bp VNTR* polymorphism in the *MAO-A* promoter region on incidences of depressive symptoms in late-reproductive-age women.
2. The relationships between hormonal factors such as AMH and FSH levels and incidences of depressive symptoms in late-reproductive-age women.

## 2. Results

The average age of the women was 42.28 ± 4.54 years. Most (74.86%) had completed third-level education; 22.83% had completed second-level education; 2.2% has completed vocational education; and 0.29% had completed only primary education. The majority of the women lived in cities with a population of more than 100,000 residents (72.54%); 11.85% and 2.89% of the participants lived in rural areas and towns of up to 10,000 residents, respectively; the remainder (12.72%) lived in towns with no more than 100,000 residents. Three quarters of the participants in the study had life partners (74.86%). Greater than half of the respondents (95.55%) were professionally active. According to the BDI, as many as 282 (81.74%) of the women did not show any depressive symptoms; however, 43 (12.46%) of them had minor, 12 (3.48%) had moderate, and eight (2.32%) had severe depressive symptoms. The participants of the study were divided into two groups: one consisted of those women lacking depressive symptoms, and the other included all other women—that is, those with minor, moderate, and severe depressive symptoms.

Analysis of the data demonstrated that the average AMH level was equal to 2.06 **±** 2.16 ng/mL, the average FSH level was 9.29 **±** 11.17mlU/mL, and the average BDI score was 6.38 **±** 6.61 (Table 1). Analysis of AMH levels with regard to the severity of depressive symptoms, as gauged by the BDI, demonstrated that the highest average AMH levels of 2.61 ± 3.01 ng/mL were noted in women with moderate depressive symptoms, while the lowest (1.95 **±** 2.08 ng/mL) were seen in women lacking depressive symptoms (Table 2). There was no statistically significant relationship between AMH level and depressive symptoms (*p* > 0.05) (Table 3).

**Table 1:** Basic statistics of AMH and FSH levels and severity of depressive symptoms according to the BDI.

Basic Statistics	N	$\bar{X}$ ± SD	Min–Max	Q1–Q3	Me
**BDI—total**	345	6.38 ± 6.61	0.00–40.00	1.00–9.00	5.00
**AMH (ng/ml)**	347	2.06 ± 2.16	0.15–11.59	0.46–2.80	1.33
**FSH (mIU/ml)**	347	9.29 ± 11.17	2.33–115.00	5.11–8.41	6.23

N—number of participants; $\bar{X}$ —arithmetic mean; SD—standard deviation; Q1—upper quartile; Q3—lower quartile; Me—median.

**Table 2:** Basic statistics of AMH (ng/mL) with regard to severity of depressive symptoms according to the BDI for N = 345.

BDI	N (%)	$\bar{X}$ ± SD	Min–Max	Q1–Q3	Me
No depressive symptoms	282 (81.74)	1.95 ± 2.08	0.15–11.14	0.42–2.63	1.31
Mild depressive symptoms	43 (12.46)	2.59 ± 2.51	0.15–11.59	0.73–3.69	1.87
Moderate depressive symptoms	12 (3.48)	2.61 ± 3.01	0.15–9.23	0.21–4.46	1.39
Severe depressive symptoms	8 (2.32)	2.10 ± 1.72	0.37–5.39	0.82–3.07	1.65
**Total**	**345 (100)**	**2.06 ± 2.17**	**0.15–11.59**	**0.46–2.76**	**1.32**

N—number of participants; $\bar{X}$ —arithmetic mean; SD—standard deviation; Q1—upper quartile; Q3—lower quartile; Me—median.

**Table 3:** AMH level (ng/mL) with regard to depressive symptoms according to the BDI.

BDI	N	$\bar{X}$ ± SD	Min–Max	Q1–Q3	Me	z	*p*
**No depressive symptoms**	282	1.95 ± 2.08	0.15–11.14	0.42–2.63	1.31	−1.76	n.s.
**Depressive symptoms**	63	2.53 ± 2.50	0.15–11.59	0.60–1.87	1.87		

z—Mann-Whitney U test statistics; *p*—significance level for z statistics; n.s.—non*-*significant.

### 2.1. The 44-bp VNTR Polymorphism in the 5HTT (SLC 6A4) Promoter Region and the 30-bp VNTR Polymorphism in the MAO-A Promoter Region vs. Depressive Symptoms

There were no statistically significant differences in the distributions of genotypes or the frequency of alleles of the *44-bp VNTR* polymorphism in the *5HTT (SLC 6A4)* promoter region or of the *30-bp VNTR* polymorphism in the *MAO-A* promoter region and depressive symptoms according to the BDI (Table 4 and Table 5). 

**Table 4:** Distribution of genotypes and frequency of alleles of the *44-bp VNTR* polymorphism in the *5HTT (SLC 6A4)* promoter region *vs.* depressive symptoms (no depressive symptoms *vs.* mild, moderate and severe depressive symptoms) according to the BDI (N = 343).

BDI	N	Genotype			χ^2^	*p*	Allele		χ^2^	*p*
		s/s n(%)	l/s n(%)	l/l n(%)			s n/(%)	l n(%)		
**Depressive symptoms**	63	8	34	21	1.4	n.s.	50	76	0.2	n.s.
		(12.7)	(54.0)	(33.3)			(39.7)	(60.3)		
**No depressive symptoms**	280	41	128	111			210	350		
		(14.6)	(45.7)	(39.6)			(37.5)	(62.5)		

n—number of participants in genotypic subgroup; χ^2^—Pearson’s chi-square test statistics; *p*—level of significance for χ^2^; n.s.—non*-*significant.

**Table 5:** Distribution of genotypes and frequency of alleles of the *30-bp VNTR* polymorphism in the *MAO-A* promoter region *vs.* depressive symptoms (no depressive symptoms *vs.* mild, moderate and severe depressive symptoms) according to the BDI (N = 343).

BDI	N	Genotype			χ^2^	*p*	Allele		χ^2^	*p*
		3/3 n(%)	3/4 n(%)	4/4 n(%)			3 n(%)	4 n(%)		
**Depressive symptoms**	62	7	29	26 (41.9)	0.3	n.s.	43	81	0.04	n.s.
		(11.3)	(46.8)				(34.7)	(65.3)		
**No depressive symptoms**	281	38	124	119			200	362		
		(13.5)	(44.1)	(42.4)			(35.6)	(64.4)		

n—number of participants in genotypic subgroup; χ^2^—Pearson’s chi-square test statistics; *p*—level of significance for χ^2^; n.s.—non*-*significant.

### 2.2. An Attempt to Define the Model 

AMH levels negatively correlated with age and FSH levels; this was a statistically significant but weak correlation (in both cases *p* < 0.05). Increases in age and FSH levels were accompanied by decreases in AMH level. The severity of depressive symptoms according to the BDI did not statistically significantly correlate with AMH levels (*p* > 0.05) (Table 6). Regression analysis of the AMH level was performed. It was assumed that the AMH level depends on age and FSH level, but the adjustment of this model to the variables (FSH and age) was very low (Table 7). 

**Table 6:** Pearson’s linear correlation between AMH level and severity of depressive symptoms according to the BDI, FSH level and age of the women.

Parameters	N	r/R *	*p*
AMH level and depressive symptoms according to the BDI-total	345	0.05	n.s.
AMH level *vs.* FSH level	345	−0.2168	0.000
AMH level *vs.* age	345	−0.2492	0.000
AMH level *vs.* BDI-groups	345	0.092 *	n.s.

r—Pearson’s rank correlation coefficient; R—Spearman’s rank correlation coefficient; *p*—level of significance for r; n.s.—non*-*significant; *—the use of R.

**Table 7:** Results of regression analysis of AMH level.

Regression Analysis	B	Standard Error	*t* (342)	*p*
**Absolute term**	6.459	1.070	6.039	0.000000
**FSH**	−0.031	0.010	−2.947	0.003433
**age**	−0.097	0.026	−3.786	0.000181

## 3. Discussion

There is abundant scientific evidence that the risk of depressive symptoms in women significantly increases with age and change in menopausal status. The influence of a decline in sex hormone levels during perimenopause on the development of depressive symptoms has so far not been fully elucidated.

Perimenopausal depressive disorders may also be determined by genetic factors, which cause abnormalities in the functioning of the monoaminergic system. It is believed that the functioning of the monoamine neurotransmitter system is mainly associated with the *MAO-A* inhibitors and the *5HTT* gene. The occurrence of depressive disorders depends on the type of these gene polymorphisms. 

In two longitudinal studies, the severity of depressive symptoms during the late reproductive period, the early perimenopause, the late perimenopause, and the postmenopause was analyzed. The study carried out by Bromberg *et al.* on a group of 2885 women showed that the risk of depressive symptoms during early and late perimenopause, as well as during postmenopause, was higher than in the late reproductive period [25]. Identical observations were made by Freeman *et al.*, who studied a group of 436 women. These authors also noted that the risk of depressive symptoms was higher in the late perimenopausal period than in the early perimenopause [3]. Their later research on a group of perimenopausal women showed that the number of women with CES-D scores ≥ 16 (Center for Epidemiologic Studies Depression Scale, CES-D) increased by a factor of 4.3 in the perimenopausal period, as compared to the late reproductive period [10]. A study of 630 postmenopausal Polish women demonstrated depressive symptoms according to the BDI in 29.2% of the participants; mild symptoms in 18.6%; moderate symptoms in 7.1%; and severe symptoms in 3.5% [26]. In the present study, the percentage of late-reproductive-age women with depressive symptoms was lower at 18.26%, which included 12.46% with mild symptoms, 3.48% with moderate symptoms, and 2.32% with severe symptoms.

Two other large-scale longitudinal studies compared individuals with a clinical diagnosis of depression based on a structured clinical interview. The research conducted within the SWAN project, which involved a multiracial and multiethnic group, revealed that depression was more often diagnosed in perimenopause and postmenopausal periods than before menopause [27]. These results were confirmed by the study of Cohen *et al.* on a group of 460 women [28]. These two studies demonstrated that the rate of major depressive episodes doubled in the perimenopausal period in comparison with the late reproductive period. In the SWAN study, the rate of incidence of depression in the postmenopausal period was four times higher than in the late reproductive period. 

Our results indicate that depressive symptoms were noted in 63 (18.26%) late-reproductive-age women (with an average age of 42 years). Potentially, this number may considerably increase with a change in menopausal status. 

Morphometric research on human ovaries suggest that the diminished stock of resting primordial follicles and a decline in the number of small growing follicles are related to age [29]. AMH is only produced by these small growing follicles; the aging process may therefore lead to a drop in the level of this hormone in the body. It is produced by granular cells located in preantral and small antral ovarian follicles. Ovarian AMH production is probably modulated by the degree of gonad development, and increases from a barely detectable level immediately after birth to a higher, though still subtle, level after puberty [30]. AMH production begins around week 36 of female fetal life and ends during menopause [31]. The precise role that AMH plays in the physiology of adult women is not yet fully understood. Nevertheless, it is believed that it may be crucial to determine the level of this hormone as a marker of fertility and ovarian reserve and as a prognostic factor of the premature cessation of ovarian function [30].

Recent studies unanimously suggest that AMH may be a more sensitive predictor of ovarian status than other, more commonly used, markers. Some authors report that the levels of serum AMH on the third day of the menstrual cycle progressively decrease with age, becoming undetectable in the postmenopausal period [32,33]. Thus, AMH concentration in peripheral blood may serve as a valuable means of monitoring the relative degree of follicular depletion that results from aging [30].

In their study carried out among normally ovulating women, de Vet *et al.* obtained results pointing to a significant decline in serum AMH concentrations over time. During their first visit, women at the average age of 29 ± 4 years had a median AMH level of 2.1 ng/mL (0.1–7.4). The second part of the study was carried out about 2.6 years after the first visit. The average age of the women was 32 years. The average AMH concentrations decreased, with a new median value of 1.3 ng/mL (0.0–5.0) in this period (*p* < 0.001). At the same time, significant changes were not observed in the levels of other markers of ovarian aging, such as serum FSH (6.0 mIU/mL *vs.* 5.8 IU/L), inhibin B (112 pg/L *vs.* 110 pg/L), and E_2_ (151 pmol/L *vs.* 161 pmol/L), as well as the number of antral follicles (invariably 14) [33]. A study conducted in Turkey demonstrated that the average serum AMH level in healthy 30-year-old women was 4.2 ng/mL [34]. 

In their research on regularly menstruating late-reproductive-age women with an average age of 41.47 years, Freeman *et al.* demonstrated that the median AMH level was 0.68 ng/mL (0.10–7.80 ng/mL). It was observed that 49% of patients reached natural menopause during the next 14 years. The average time between menopause and the first determination of AMH level was 9.81 years. Interesting results were obtained when the whole study group was divided into quartile subgroups in relation to serum AMH levels. The authors emphasized the relationship between AMH levels and the age of menopause. In the group with the lowest levels of this hormone (<0.20 ng/mL), menopause occurred within an average of 6.09 years and affected 61% of women. In the group with the highest AMH levels (>1.50 ng/mL), menopause occurred within 12.88 years and affected only 25% of the group; the remainder had not reached menopause by the end of the study. Over the 14 years of the study, in 73% of the women, AMH levels became undetectable. The average duration between this moment and menopause was 5.97 years. These findings clearly show that AMH and age are strong, independent predictors of the age of onset of menopause [35]. More recent studies also place emphasis on the role of AMH as a predictive factor in the age of menopause [36,37] and the time to menopause from the moment when AMH concentrations drop below a detectable level [38]. In this study, conducted on a group of 347 regularly menstruating women not reporting any health problems, the median AMH concentration was 1.33 ng/mL, despite the fact that the group was significantly older (42 years on average) than in the study of de Vet *et al.* [32]. Freeman [35], who analyzed a group of women of similar age (41.47 years), also noted that the average AMH level was lower than that obtained in the study described in this article. Freeman *et al.* carried out a cohort study with six assessment periods over 4 years, noting changes in sex hormones in the late reproductive and transitional periods; they demonstrated that the likelihood of depressive symptoms decreased for women with a rapidly increasing follicle-stimulating hormone profile. What is more, they noticed that increasing estradiol levels were significantly associated with depressive symptoms [3]. In the study presented here, special attention has been paid to the relationship between AMH levels and the severity of depressive symptoms. The authors did not observe any significant connection between these two elements, although this finding may be connected with the relatively small number of women with depressive symptoms in the study. Nevertheless, given the results of the study of Freeman *et al.* [3,35], it may be an interesting area for further research. 

It has been documented that a decreased level of serotonin may be responsible for mood disorders, and the *5HTT (SLC6A4)* gene that encodes the serotonin transporter is one of the most frequently studied genes in depressive disorders [39,40]. Many authors suggest that the presence of the “s” allele of the *5HTT* polymorphism (the *5-HTT* “s” variant), combined with adverse effects of environmental factors, increases the probability of depression or mood disorders as a result of stressful life events [41,42,43,44,45,46,47,48]. 

The research conducted by Hauser *et al.* demonstrated that the presence of the “s” allele may predispose to affective disorders. They showed that the s/s genotype is more common in patients with unipolar (*p* = 0.001) and bipolar (*p* = 0.003) affective disorders than in healthy individuals. This genotype was mostly found in men with unipolar affective disorder [49]. On the other hand, Grochans *et al.*, in their study of postmenopausal women, did not observe any significant associations between the genotype distribution and the allele frequency of the 44-bp polymorphism in the 5HTTLPR promoter region and depressive symptoms. They did find, however, a relationship between the severity of climacteric symptoms and the allele frequency of the polymorphism in the 5-HTT “s” variant [26].

*MAO-A* plays a key role in the degradation of monoamines, such as serotonin and noradrenalin, which contributes to the development of depression [20]. However, there are no clear conclusions from the analysis of the relationship between *MAO-A* polymorphism and the tendency to affective disorders. Some authors claim that being a carrier of the high-activity *MAO-A* polymorphism can increase the risk of developing depression [50,51]. A similar relationship has been demonstrated between the presence of this genotype and the frequency of suicidal attempts [52,53]. 

Our study, conducted on healthy late-reproductive-age women, did not reveal any statistically significant differences in the genotype distribution or the allele frequency of the *44-bp VNTR* polymorphism in the *5HTT (SLC 6A4)* promoter region, the *30-bp VNTR* polymorphism in the *MAO-A* promoter region, and the severity of depressive symptoms. It should be emphasized, however, that the study involved only healthy women without a clinical diagnosis of depression. 

The studies on women performed by Schulze [50] and Rivera *et al.* [54] show that only the presence of high-activity *MAO-A* alleles contributes to the development of symptoms of severe depression [50]. Another study on 332 postmenopausal women with climacteric symptoms demonstrates the relationship between the c.1460C > T polymorphism of *MAO-A* gene polymorphism and the occurrence of depressive symptoms [55]. Completely different results were reported by Brummett *et al.*, who suggested a relationship between the development of depression and low-activity alleles [56]. Both in our study carried out among late reproductive-age women, and in the study of 630 postmenopausal Polish women, the Beck Depression Inventory was used. The analysis did not confirm any differences in the frequency of genotypes and alleles of the 30-bp VNTR polymorphism in the *MAO-A* promoter region and the occurrence of depressive symptoms between the two analyzed groups [26]. 

The ability to precisely determine the age of menopause may be very useful in clinical practice, and especially in counseling women on their reproductive plans. It may also contribute to early prophylaxis of and treatment for the adverse effects of menopause, including depressive disorders.

An examination of the literature shows that AMH and FSH are good indicators of ovarian function. We have therefore made an attempt to assess the influence of changes in the levels of these hormones on the severity of depressive symptoms experienced by late-reproductive-age women, with regard to their predisposition to depressive disorders due to the presence of certain *5HTT* and *MAO-A* gene polymorphisms. The study did not confirm the relationship between the investigated variables. 

## 4. Limitations

In our research, the serum AMH levels in late-reproductive-age women were determined only once. It would be useful to repeat the tests in order to observe the decline in AMH level. The authors intend to continue the research 5, 10, and 15 years after this first study in order to determine the age of natural menopause in the study group. 

This study did not demonstrate the relationship between serum AMH level and depressive symptoms. Understanding of these issues could be extended by the studies on selected women with a clinical diagnosis of depressive disorders. 

The study group consisted of late-reproductive-age women who were at a significantly lower risk of depressive symptoms than perimenopausal and postmenopausal women. It might be interesting to continue the analysis with women whose menopausal status puts them at a high risk of developing depressive symptoms. 

Although the limitations of the present study do not allow us to extend our conclusions to the general population, it has the potential to provide interesting findings in the field requiring further research. It is necessary to conduct further research to confirm or reject the hypothesis proposed here.

## 5. Conclusions

1. The presence of the s/s genotype of the *44-bp VNTR* polymorphism in the *5HTT (SLC 6A4)* promoter region and the 3/3 genotype of the *30-bp VNTR* polymorphism in the *MAO-A* promoter region does not contribute to the development of depressive symptoms in late-reproductive-age women.
2. A relationship was not confirmed between AMH level and depressive symptoms in a group of healthy late-reproductive-age women.
3. AMH levels correlate negatively with FSH levels and age, which confirms that AMH can be regarded as a good indicator of ovarian reserve.

## 6. Material and Methods

### 6.1. Subjects

The study involved 347 healthy late-reproductive-age women from northwest Poland. Subjects were recruited through community advertisements. The criteria for inclusion in the study were regular menstruation, normal smear test results, normal mammogram or breast ultrasound results, normal blood pressure, no alcohol abuse, no cigarette smoking, no current or past history of endocrine disorders (such as diabetes or thyroid diseases), no current or past history of neoplastic diseases, and no current or past history of psychiatric treatment.

The criteria for exclusion from the study were abnormal smear test results, abnormal mammogram or breast ultrasound results, diagnosis of thyroid diseases or diabetes, diagnosis of neoplastic disease, diagnosis of mental disease, and addictions.

According to the STRAW + 10 staging system, the patients included in the study were at the ‒3b stage of STRAW + 10—that is in the late reproductive stage. The ‒3b stage represents the period in which menstrual cycles remain regular without change in length or early follicular phase FSH levels, but AMH and antral follicle counts are low [1].

All subjects gave their informed consent for inclusion in the study. The study was conducted in accordance with the Declaration of Helsinki, and the protocol was approved by the Bioethical Commission of the Pomeranian Medical University in Szczecin (permission number KB-0012/12/12). 

### 6.2. Assessments

The first stage of the study was based on a diagnostic survey performed using a standard research instrument, namely the Beck Depression Inventory (BDI), for the assessment of depressive symptoms [57]. A statistical analysis was performed on women lacking depressive symptoms (0–11 points in the BDI) and women with minor (12–19 points), moderate (20–25 points), or severe depressive symptoms (over 26 points). Women with Axis I mental disorders, according to the ICD-10 classification, were excluded from the analysis by means of a PRIME-MD questionnaire and a psychiatric examination [58]. 

The second stage of the study was based on genetic tests. DNA was isolated from whole blood by the salting-out method of Miller [59]. Polymerase chain reaction (PCR) was used to identify DNA polymorphisms. The aim of the analysis was to amplify the fragment consisting of 2–5 repetitions of the *30-bp VNTR* polymorphism in the *MAO-A* promoter region. The following primer sequences were used: *MAO-A-F, 5’-CCC-AGG-CTG-CTC-CAG-AAA-3’*, and *MAO-A-R, 5’-GGA-CCT-GGG-CAG-TTG-TGC-3’*. The PCR consisted of an initial denaturing step at 95 °C for 3 min, followed 8 by 34 cycles of denaturing at 94 °C for 40 s, annealing at 57 °C for 35 s, and polymerization at 72 °C for 50s, with a final elongation step at 72 °C for 10 min.

The sizes of the amplified fragments were as follows: 239, 209, 226, and 269 bp. In the 5HTT polymorphism analysis, the fragment—including the 44-bp ins/del in the regulatory sequence (the presence or lack of 44-bp)—was amplified. The following primer sequences were used: *HTT-F*, *5’-GGC-GTT-GCC-GCT-CTG-AAT-GC-3’*, and *HTT-R, 5’-GAG-GGA-CTG-AGC-TGG-ACA-ACC AC-3’*. The PCR consisted of an initial denaturing step at 94 °C for 5 min, followed by 30 cycles of denaturing at 94 °C for 55 s, annealing at 55 °C for 50 s, and polymerization at 72 °C for 60s, with a final elongation step at 72 °C for 10 min. The sizes of the amplified fragments were 484 and 528 bp. The PCR products were electrophoresed on 3% agarose gel, which was followed by ethidium bromide staining to detect the alleles [60,61]. 

FSH and AMH levels were determined as the third stage of the study. Venous blood samples were collected from women in the follicular phase of the menstrual cycle using a closed system (Vacutainer), after the women gave their consent to this procedure. The blood was drawn in the treatment room and delivered to the laboratory in accordance with the relevant rules and procedures. The levels of FSH and AMH were determined in a laboratory accredited with ISO 9001:2008 quality certification. The FSH ranges accepted in the study as normal were FSH follicular levels—that is, 3.5–12.5 mIU/ml. 

### 6.3. Statistical Analyses

Statistical analysis was performed using Statistica 7.1 PL. Pearson’s chi-squared independence test was applied to verify the null hypothesis regarding the independence of the variables. Spearman’s rank *R* correlation coefficient was used to identify and to test the strength of a relationship between the ordinal variables. The significance level was set at *α* = 0.05. The Mann–Whitney *U*-test was used to assess the relationship between AMH levels and depressive symptoms according to the BDI. The power calculated for all of the genetic tests exceeded 0.95.

A stepwise regression method with a process of elimination was applied to find elements having the strongest influence on the AMH level. During the analysis, explanatory variables unrelated to the response variables were rejected. The model with the corrected R2, explaining the variance in the independent variable, was presented. Explanatory variables are selected *a priori* by means of stepwise regression, in the following way:

1. We estimated econometric models with one explanatory variable. From among potential explanatory variables, we chose the one corresponding to the highest absolute value of Student’s *t*-statistic. If this variable parameter is statistically significant, we pass to the second stage. Otherwise, none of the candidate variables explains the dependent variable.
2. We estimate m-1 models with two explanatory variables, one of which was selected in the preceding step. Again, from among the candidate variables, we choose the one with the highest absolute value of Student’s *t-*statistic. From the created model, we remove the variables that have no significant impact on the phenomenon analyzed.
3. If none of the remaining candidate variables can be added to the model, the estimated equation has the optimal combination of explanatory variables [62].
